# In vitro inactivation of SARS-CoV-2 using a povidone-iodine oral rinse

**DOI:** 10.1186/s12903-022-02082-9

**Published:** 2022-02-25

**Authors:** Manjunath Shet, Jonna Westover, Rosa Hong, David Igo, Marc Cataldo, Sailaja Bhaskar

**Affiliations:** 1 Imbrium Therapeutics L.P., Stamford, CT USA; 2 The Institute for Antiviral Research, Utah State University, Logan, UT USA; 3 Avrio Health L.P., New York, NY USA; 4 Purdue Pharma L.P., Stamford, CT 06901 USA

**Keywords:** Anti-infective agents, Coronavirus, Povidone-iodine, SARS-CoV-2, Virus inactivation

## Abstract

**Background:**

Healthcare professionals, especially dentists and dental hygienists, are at increased risk for contracting severe acute respiratory syndrome coronavirus 2 (SARS-CoV-2) through air-borne particles and splatter. This study assessed the in vitro virucidal activity of 0.5% (w/v) povidone-iodine (PVP-I) oral rinse against SARS-CoV-2 to demonstrate its utility as a professional oral rinse.

**Methods:**

A 0.5% (w/v) PVP-I oral rinse formulation, placebo oral rinse, and positive (70% [v/v] ethanol and water) and negative (water) controls were assessed using the time-kill method. SARS-CoV-2 was propagated in Vero 76 host cells. Following neutralization validation, triplicate tests were performed for each test formulation and virucidal activity measured at 15, 30, and 60 s and 5 min.

**Results:**

The 0.5% (w/v) PVP-I oral rinse demonstrated effective in vitro virucidal activity against SARS-CoV-2 as early as 15 s after exposure; viral titer was reduced to < 0.67 log_10_ 50% cell culture infectious dose (CCID_50_)/0.1 mL (log_10_ reduction of > 4.0) at 30 s, whereas the placebo oral rinse reduced the SARS-CoV-2 viral titer to 4.67 and 4.5 log_10_ CCID_50_/0.1 mL at the 15- and 30-s time points, with a log_10_ reduction of 0.63 and 0.17, respectively. No toxicity or cytotoxic effects against Vero 76 host cells were observed with the 0.5% (w/v) PVP-I oral rinse; positive and negative controls performed as expected.

**Conclusions:**

In vitro virucidal activity of 0.5% (w/v) PVP-I oral rinse against SARS-CoV-2 was demonstrated. Rapid inactivation of SARS-CoV-2 was observed with 0.5% (w/v) formulation with a contact duration of 15 s. Clinical investigations are needed to assess the effectiveness of PVP-I oral rinse against SARS-CoV-2 in dental practice.

## Background

Since the emergence of the novel severe acute respiratory syndrome coronavirus 2 (SARS-CoV-2) toward the end of 2019, global daily cases peaked at almost 1.5 million in December 2020, and almost 18,000 daily deaths were reported globally in January 2021 [1]. This new and highly transmissible SARS-CoV-2 has impacted all levels of society; however, healthcare professionals are at a higher risk of contracting the virus as a result of their prolonged and repeated exposure to infected and highly contagious patients.

The principal route of transmission of SARS-CoV-2 is via respiratory droplets and the upper respiratory tract. However, an emerging hypothesis suggests a vascular route of transfer of SARS-CoV-2 from the oral cavity to the respiratory system [2]. The virus can also spread through the conjunctiva [3], and high viral loads of SARS-CoV-2 have been detected in the nasopharynx and oropharynx of symptomatic as well as asymptomatic individuals [4].

Despite the use of personal protective equipment (PPE), certain healthcare professionals such as dentists and dental hygienists may be at a higher risk of exposure to the virus than others because aerosols and splatter released during dental procedures present an environment with a high risk of contamination through air-borne particles [5]. Consequently, the American Dental Association (ADA) interim guidelines (October 2020) and the Centers for Disease Control and Prevention (CDC) have proposed changes to dental procedures and preprocedural oral rinse [6, 7]. Although there is currently no published evidence regarding the clinical effectiveness of preprocedural mouth rinses (PPMRs) to prevent SARS-CoV-2 transmission, PPMRs with an antimicrobial product such as chlorhexidine gluconate, essential oils, povidone-iodine (PVP-I), or cetylpyridinium chloride may reduce the level of oral microorganisms in aerosols and spatter generated during dental procedures [7]. Furthermore, while there currently seems to be insufficient scientific evidence to support the use of hydrogen peroxide as an oral rinse, in vitro studies and small clinical studies or case reports conducted with ex-US formulations have demonstrated effective antiviral activity of PVP-I solutions against SARS-CoV-2 [8–10]. A small-scale randomized study in SARS-CoV-2–positive patients assessing the efficacy of PVP-I, chlorhexidine gluconate, and cetylpyridinium chloride in reducing salivary SARS-CoV-2 viral load found that viral load was reduced for up to 6 h with cetylpyridinium chloride or PVP-I, whereas another in vivo test demonstrated a significant reduction in viral load for at least 3 h after PVP-I oral rinse in 50% of patients [9, 10]. In addition, the use of PVP-I formulations has been evaluated for high-risk clinical procedures involving the oropharynx and nasopharynx and in surgical practice [11–13].

PVP-I is an antiseptic agent with broad-spectrum anti-infective activity against a variety of pathogenic microorganisms, including viruses [14–17], with demonstrated in vitro virucidal activity against enveloped and nonenveloped viruses over short contact times relative to other commercially available antiseptic agents [18–21]. The efficacy and tolerability profiles of PVP-I compared with that of other agents, such as chlorhexidine gluconate, polyhexanide, and octenidine, have been well established [13, 15, 16, 22], and no resistance or cross-resistance with PVP-I has been documented in the past [13–15].

SARS-CoV-2 has profoundly altered the fundamental dynamics of clinical dentistry worldwide and there is a great need to raise awareness among dental practitioners regarding the virucidal activity of commercially available oral rinses. Strikingly, a recent study assessing practitioners’ knowledge, attitude, and practices for oral rinse use amidst the pandemic revealed that only 38.9% of participants surveyed were aware that PVP-I was more efficient in reducing coronaviruses compared with chlorhexidine-based oral rinses. Furthermore, only 33.9% were aware that 0.23% concentration of PVP-I had substantial virucidal activity against SARS-CoV, MERS-CoV, influenza virus, and rotavirus [23, 24].

The aim of this study was to investigate the in vitro virucidal activity of 0.5% (w/v) PVP-I oral rinse against SARS-CoV-2 at four different contact times to demonstrate its utility as a professional oral rinse.

## Methods

### Test formulations

A 0.5% (w/v) PVP-I oral rinse formulation (Betadine^®^ Oral Rinse, Avrio Health L.P.) was assessed for its in vitro virucidal activity against SARS-CoV-2 and compared against a placebo oral rinse, a positive control comprising 70% (v/v) ethanol, and a negative control comprising water.

### Virus strains and host cells

The SARS-CoV-2, strain USA-WA1/2020, was kindly provided by the World Reference Center for Emerging Viruses and Arboviruses at The University of Texas Medical Branch. The virus was passaged twice in Vero 76 cells (ATCC CRL-1587) to create the working stock.

### Facilities

The assays were performed at the Institute for Antiviral Research, Utah State University, Logan, Utah, USA. Standard equipment and supplies were used; calibration was performed in accordance with the standard operating procedures of the facility. This study did not include animal experiments or human subject research.

### Preparation of virus suspensions and host cells

The method has been described previously [25]. Briefly, virus strains were propagated and stored per standard procedure for the production of high-titer virus stock. The culture medium used for the virucidal assay (test medium) was Minimum Essential Medium (MEM) with 2% fetal bovine serum (FBS) and 50 μg/mL gentamicin. Host cells were maintained as monolayers in disposable cell culture labware. Before testing, these cultures were seeded onto multiwell cell culture–treated plates. For virucidal suspension testing, Vero 76 cell monolayers were grown to 80%–90% confluence.

### Neutralization validation

Neutralization validation was performed to confirm the effectiveness of the procedure in neutralizing the active virucidal component; this was performed for the positive control, negative control, the active test formulation, and the respective placebo. Neutralization was validated when virus recovery in the positive control matched that in the neutralized formulation.

### Virucidal suspension test

Triplicate tests were set up for each test formulation. Each test tube contained 50 μL of the test formulation mixed with 50 μL of the high-titer virus suspension. Samples were incubated at 22 ± 2 °C for designated exposure times of 15 s, 30 s, 60 s, and 5 min. At the designated exposure time, samples were neutralized by performing a 1/10 dilution in MEM + 2% FBS + 50 μg/mL gentamicin. Subsequently, samples were pooled and serially diluted 1/10 using eight log_10_ dilutions in the test medium before being added to quadruplicate columns of 96-well plates seeded with monolayers of 80%–90% confluent Vero 76 cells. The plates were incubated until a maximum cytotoxic effect was observed in the control wells, and cytotoxicity was recorded as a binary result. Negative controls were run, which were identical to the test formulation runs, except that water was substituted for the 0.5% (w/v) PVP-I oral rinse. Toxicity controls were run in four additional wells of Vero 76 cells, with two wells being infected with the virus at each dilution to serve as neutralization controls, ensuring that the residual sample in the titer assay plate did not inhibit growth and detection of the surviving viruses. The plates were incubated at 37 ± 2 °C in a 5% carbon dioxide (CO_2_) atmosphere for 5 days. Each well was then scored for the presence or absence of infectious virus.

### Analytical methods

Viral titers were reported as log_10_ of the 50% titration end point for infectivity, expressed as the 50% cell culture infectious dose (CCID_50_), and were calculated using the Reed-Muench method [26]. The log_10_ of infectivity reduction, or log_10_ reduction value (LRV), was calculated using the following formula: LRV = (log_10_ CCID_50_ of the negative control) − (log_10_ CCID_50_ of the virucidal suspension test).

Viral titer was calculated as the difference in log_10_ CCID_50_/0.1 mL between the negative control and the test formulation; this was done at the designated exposure times (i.e., 15 s, 30 s, 60 s, and 5 min).

## Results

All test formulations were validated for neutralization across triplicates (data not shown). The 0.5% (w/v) PVP-I oral rinse demonstrated effective in vitro virucidal activity against SARS-CoV-2 as early as 15 s after exposure, with a reduction of viral titer to 2.5 and < 0.67 log_10_ CCID_50_/0.1 mL (log_10_ reduction of 2.8 and > 4.0) at 15 and 30 s, respectively, under test conditions, whereas the placebo oral rinse reduced the SARS-CoV-2 viral titer to 4.67 and 4.5 log_10_ CCID_50_/0.1 mL at the 15- and 30-s time points, with a log_10_ reduction of 0.63 and 0.17, respectively (Table 1). The efficacy of the positive and negative controls was also evaluated at exposure times of 15, 30, and 60 s and 5 min. The negative control for the 0.5% (w/v) PVP-I oral rinse formulation showed titer levels of 5.3 log_10_ CCID_50_/0.1 mL at 15 s and 4.67 log_10_ CCID_50_/0.1 mL at 5 min. The positive control demonstrated effective titer reduction to 1.3 and < 0.67 log_10_ CCID_50_/0.1 mL, with log_10_ reductions in infectivity of 4.0 and > 4.0 at the 15- and 30-s time points, respectively. No toxicity was observed with the 0.5% (w/v) PVP-I oral rinse, and the positive and negative controls performed as expected. No cytotoxic effects against the Vero 76 host cells were observed with the 0.5% (w/v) PVP-I oral rinse.

**Table 1:** In vitro virucidal activity of 0.5% (w/v) PVP-I oral rinse formulation against SARS-CoV-2

	Tested concentration (%)	Incubation time	Virus titer^a^	LRV^b^
0.5% (w/v) PVP-I oral rinse	50	15 s	2.5	2.8
Placebo oral rinse	50		4.67	0.63
Positive control	50		1.3	4.0
Negative control	NA		5.3	–
0.5% (w/v) PVP-I oral rinse	50	30 s	< 0.67	> 4.0
Placebo oral rinse	50		4.5	0.17
Positive control	50		< 0.67	> 4.0
Negative control	NA		4.67	–
0.5% (w/v) PVP-I oral rinse	50	60 s	1.0	3.67
Placebo oral rinse	50		5.0	0
Positive control	50		< 0.67	> 4.0
Negative control	NA		4.67	–
0.5% (w/v) PVP-I oral rinse	50	5 min	< 0.67	> 4.0
Placebo oral rinse	50		4.67	0
Positive control	50		< 0.67	> 4.0
Negative control	NA		4.67	–

Samples were incubated at 22 ± 2 °C for designated exposure times of 15 s, 30 s, 60 s, and 5 min

Abbreviations: CCID_50_, 50% cell culture infectious dose; LRV, log_10_ reduction value; NA, not applicable; PVP-I, povidone-iodine; SARS-CoV-2, severe acute respiratory syndrome coronavirus 2; w/v, weight per volume

^a^Log_10_ CCID_50_ of virus per 0.1 mL. The assay lower limit of detection is 0.67 log_10_ CCID_50_/0.1 mL

^b^LRV is the reduction of the virus compared with that of the negative control

## Discussion

Several in vitro studies suggest that antiseptics may reduce the viral load of SARS-CoV-2 or other coronaviruses; however, limited in vivo evidence for oral antiseptics currently exists. Furthermore, available in vivo studies have several limitations such as small sample sizes and lack of suitable control groups, which leads to conflicting or inconclusive efficacy results [27]. In addition, accurate collection and measurement/quantification of SARS-CoV-2 viral load through cycle count amplification can lead to imprecise results. The current study thus set out to demonstrate in vitro, the virucidal activity of the PVP-I oral rinse at a concentration of 0.5% (w/v) PVP-I and as early as 15 s after being challenged with SARS-CoV-2. The observed log_10_ reduction in the viral titer of > 4.0 at 30 s after the contact time and lasting up to 5 min is indicative of rapid and prolonged viral inactivation. Intrinsic variance of the assay due to visual determination of the cytopathic effect is the likely cause for the LRV of 3.67 observed at 60 s. In agreement with these findings, a recent study has also shown that oral rinses containing povidone-iodine as the active compound reduce viral infectivity to up to three orders of magnitude to background levels [28].

Physical intervention, as an adjunct to supplement the use of PPE, to reduce viral transmission during the current SARS-CoV-2 pandemic and beyond may be a necessary step to ensure a safe environment for patients and healthcare workers alike. Oral rinses that can reduce the viral load in preprocedural settings, especially those where patients’ mouths and noses are exposed, may be a critical component in the prevention of transmission. Indeed, an observational study on the tolerability and usability of 0.5% (w/v) PVP-I gargles and nasal drops in 6,692 patients attending ENT consultations as a prerequisite examination reported these formulations to be feasible and useable and that they provide a needed benefit in preventing transmission between patients and healthcare workers [29]. In addition, the in vivo application of PVP-I has also been proposed to reduce viral load in otorhinolaryngology surgical practices. The use of 0.5% (w/v) PVP-I has several advantages such as its ease of preparation, cost-effectiveness, safe use, and its potential to reduce viral titers of SARS-CoV-2 [30]. Currently available alternatives to the PVP-I oral rinse comprise chlorhexidine gluconate rinse, which has been shown to have weak virucidal activity [31], and hydrogen peroxide, which, although recommended by the ADA, has insufficient supporting scientific evidence to be classified as effective for the prophylaxis of SARS-CoV-2 infection [6, 7, 31]. In addition, a systematic review reported that there is currently no available clinical evidence to support the use of nasal sprays or oral rinses to protect against SARS-CoV-2 transmission among healthcare workers but indicated that three studies (including two phase 2 randomized controlled trials) are underway to assess the level of protection that can be expected from these modes of antiseptic administration [32]. Indeed, results from a recent randomized clinical trial assessing the nasopharyngeal application of PVP-I solutions to reduce the viral load of patients with non-severe COVID-19 symptoms revealed that the use of PVP-I did not influence changes of viral ribonucleic acid (RNA) quantification over time. In addition, the study also reported a greater mean relative difference in viral titers between baseline and day 1 for participants in the intervention group (75%) compared with those in the control group (32%) [33].

This study adds to the emerging evidence that demonstrates the in vitro antiviral effectiveness of PVP-I oral rinse as early as 15 s against SARS-CoV-2 at a concentration of 0.5% (w/v). These findings suggest that the use of preprocedural PVP-I oral rinses as an adjunct to PPE for patients and healthcare providers is a viable option [6, 7], which is supported by the inclusion of PVP-I oral rinse into the World Health Organization (WHO) R&D Blueprint for COVID-19 Experimental Treatments [34].

## Conclusion

This study demonstrated the in vitro virucidal activity of a PVP-I (0.5% w/v) oral rinse against SARS-CoV-2 using the time-kill method*.* Rapid inactivation of SARS-CoV-2 was observed at a concentration of 0.5% (w/v), with a contact duration of as early as 15 s. Following these promising results, clinically focused investigations are needed to assess the effectiveness of PVP-I oral rinse in the dental practice setting, perhaps including various strains or variants of SARS-CoV-2.

## Data Availability

All data generated and analyzed during this study are available from the corresponding author on reasonable request.

## Abbreviations

ADA: American Dental Association
CCID_50_: 50% Cell culture infectious dose
CDC: Centers for Disease Control and Prevention
CO_2_: Carbon dioxide
FBS: Fetal bovine serum
LRV: Log_10_ reduction value
MEM: Minimum essential medium
MERS-CoV: Middle East respiratory syndrome-related coronavirus
NA: Not applicable
PPE: Personal protective equipment
PPMRs: Preprocedural mouth rinses
PVP-I: Povidone-iodine
RNA: Ribonucleic acid
SARS-CoV-2: Severe acute respiratory syndrome coronavirus 2
w/v: Weight per volume
WHO: World Health Organization
